# The prognostic value of the CALLY index in sepsis: a systematic review and meta-analysis

**DOI:** 10.3389/fmed.2026.1812568

**Published:** 2026-05-08

**Authors:** Chuangying Xie, Peng Sun, Min Zhang, Hong Fan, Zhenzhen Li, Xiang Tong

**Affiliations:** 1 Department of Pulmonary and Critical Care Medicine, West China Hospital, Sichuan University, Chengdu, China; 2 State Key Laboratory of Respiratory Health and Multimorbidity, Chengdu, China; 3 Health Management Center, West China Hospital of Sichuan University, Chengdu, Sichuan, China

**Keywords:** CALLY index, meta-analysis, mortality, prognosis, sepsis

## Abstract

**Background:**

The C-reactive protein-albumin-lymphocyte (CALLY) index is a composite biomarker of inflammation, nutrition, and immunity, yet its prognostic value in sepsis remains uncertain due to inconsistent evidence. This meta-analysis seeks to clarify the relationship between the CALLY index and prognosis in septic patients.

**Methods:**

A systematic search of PubMed, Embase, OVID, Web of Science, the China National Knowledge Infrastructure, the Wanfang Database, Google Scholar, and Baidu Scholar was conducted up to November 15, 2025. Studies reporting on the CALLY index’s association with mortality or its levels in sepsis survivors versus non-survivors were included. Data were pooled using standardized mean differences (SMDs), hazard ratios (HRs), and odds ratios (ORs) with 95% confidence intervals (CIs) under a random-effects model.

**Results:**

This meta-analysis incorporated a total of six studies, encompassing seven cohorts and comprising 3,848 patients with sepsis. Overall, the CALLY index did not significantly differ between survivors and non-survivors (pooled SMD = −0.22, 95% CI: −1.18 to 0.74). However, subgroup analyses revealed a marked divergence: a higher CALLY index was correlated with survival in Chinese cohorts (SMD = −1.04, 95% CI: −1.69 to −0.39), whereas it was associated with mortality in the Turkish cohort (SMD = 1.39, 95% CI: 1.02 to 1.75). Multivariate analysis further showed a significant association between a higher CALLY index and a lower mortality risk (HR = 0.48, 95% CI: 0.33–0.69). The diagnostic accuracy of the CALLY index for predicting mortality was moderate, with a pooled sensitivity of 0.59 and specificity of 0.77. Significant heterogeneity was observed across all pooled analyses.

**Conclusion:**

The CALLY index shows promise as a low-cost sepsis biomarker, but its variable link to mortality across ethnic groups requires validation in large, multinational prospective studies.

**Systematic review registration:**

Identifier: INPLASY2025120023. http://inplasy.com

## 1 Introduction

Sepsis and septic shock claim millions of lives each year globally, constituting a substantial health burden and a critical public health challenge to medical systems worldwide (1, 2). In the clinical management of sepsis, various biomarkers assist in early detection and prognostic evaluation, such as lactate, procalcitonin (PCT), C-reactive protein (CRP), albumin, and lymphocyte count (3, 4). CRP, a widely recognized acute-phase protein, is closely linked to adverse outcomes in septic patients (5). Hypoalbuminemia, frequently seen in critical illness, reflects both catabolic stress and endothelial dysfunction (6). Lymphocyte count serves as an indicator of immune competence, and sepsis-induced lymphopenia is a well-documented phenomenon that promotes immune paralysis, elevating the risk of secondary infections and death (7). Nevertheless, the utility of these individual parameters is often limited in practice due to significant inter-individual variability and confounding factors. Combining biomarkers into ratio-based indices offers a promising approach to reduce such heterogeneity by accounting for dynamic fluctuations.

The C-reactive protein-albumin-lymphocyte (CALLY) index is a composite and easily calculable score that incorporates biomarkers of inflammation, nutritional status, and immune function. Although previous studies have established its prognostic utility in various cancers (8, 9), recent evidence also suggests a role for the CALLY index in sepsis. However, findings regarding its diagnostic and prognostic value in septic patients remain inconsistent. For example, Yılmaz et al. reported in a Turkish cohort that a higher CALLY index was associated with increased sepsis-related mortality (10), while Zhang et al. observed that a lower CALLY index correlated with fatal outcomes (11). To date, no systematic review or meta-analysis has comprehensively evaluated the relationship between the CALLY index and sepsis. Therefore, we conducted a study to clarify this association and to assess the potential of the CALLY index as a practical prognostic parameter in the management of sepsis.

## 2 Methods

### 2.1 Protocol and registration

We conducted this systematic review in strict accordance with the Preferred Reporting Items for Systematic Reviews and Meta-Analyses (PRISMA) 2020 guideline. The study protocol was prospectively registered on the International Platform of Registered Systematic Review and Meta-Analysis Protocols (INPLASY) with the unique registration number INPLASY2025120023.

### 2.2 Search strategy

We conducted a comprehensive systematic literature search in PubMed, Embase, OVID, Web of Science, the China National Knowledge Infrastructure (CNKI), and the Wanfang Database to identify studies exploring the application of the CALLY index in patients with sepsis. The search encompassed all records from the inception of each database up to November 15, 2025. This search strategy was applied with the core retrieval formula constructed as follows: (“sepsis” OR “bloodstream infection” OR “bacteremia”) AND (“C-reactive protein-albumin-lymphocyte index” OR “CRP-albumin-lymphocyte index” OR “CALLY index”). For Chinese databases, the corresponding Chinese terms were used for searching. Detailed search strategies are reported in Supplementary Table S1. The literature search was conducted with restrictions to publications in English and Chinese. To minimize the risk of omission, a supplementary search was performed using Google Scholar and Baidu Scholar, reviewing the initial pages of results, and manually scanning the reference lists of relevant retrieved articles. As this meta-analysis utilized exclusively aggregated data from previously published studies, neither ethical approval nor patient consent was required.

### 2.3 Study selection

The eligibility criteria for this systematic review were established as follows: (1) original clinical investigations employing a cohort or case–control design that evaluated the utility of the CALLY index in patients with sepsis; (2) for overlapping or duplicate datasets, only the most recent and/or comprehensive publication was retained to ensure data independence; and (3) studies had to report sufficient quantitative outcomes—such as standardized mean differences (SMDs), hazard ratios (HRs), or odds ratios (ORs) with 95% confidence intervals (CIs)—to permit meta-analytic pooling of effect sizes, or to allow calculation of summary sensitivity and specificity measures. Studies were excluded if (1) essential effect size data could not be extracted or key methodological information was incomplete, or (2) they were non-original articles (e.g., reviews, letters, conference abstracts) or duplicate publications.

### 2.4 Risk of bias

To ensure a rigorous quality assessment, the included studies pertaining to the CALLY index in sepsis were evaluated with the Newcastle-Ottawa Scale (NOS). The NOS instrument judges quality based on three criteria: selection, comparability, and exposure/outcome (12). To ensure consistency, a consensus meeting involving all authors was conducted to discuss individual assessments and resolve discrepancies, thereby finalizing a score for each study. The NOS assigns a maximum of 9 points, with total scores of 0–3, 4–6, and 7–9 representing low, moderate, and high quality, respectively.

### 2.5 Data extraction

The demographic and clinical data from each eligible study were systematically extracted by two independent authors (CYX and PS) using a predefined data extraction form in Excel. Any discrepancies or uncertainties arising during the data collection process were resolved through consultation with a third author (MZ) for final arbitration. The data extraction protocol included the following items: first author, year of publication, ethnicity, age of participants, sample size, quantitative measurements of the CALLY index, HR or OR with corresponding 95% CI, as well as diagnostic performance metrics (sensitivity and specificity). For studies reporting median values along with ranges or interquartile ranges (IQR), these data were transformed into approximate means and standard deviations (SD) using the validated methods described by Wan et al. (13).

### 2.6 Statistical methods

All statistical analyses were performed using STATA 14.0 (StataCorp) and Meta-DiSC version 1.4 (Cochrane Collaboration). A random-effects model was adopted for all meta-analytical syntheses, as it methodologically accounts for between-study variation and typically yields more conservative effect estimates with a reduced risk of type I errors compared to fixed-effect models (14). We calculated SMDs with 95% CIs to compare CALLY index values between cohorts. To evaluate diagnostic performance, we computed pooled sensitivity, specificity, and diagnostic odds ratio (DOR). Between-study heterogeneity was assessed using the chi-square-based Cochran’s Q test and the I^2^ statistic. An I^2^ value greater than 50% or a *p*-value from the Q-test below 0.10 was considered indicative of substantial heterogeneity. Leave-one-out sensitivity analysis was performed to examine the influence of individual studies on the overall results, giving particular attention to those rated as low-quality or identified as potential outliers. Publication bias was evaluated visually using funnel plots and formally tested using Egger’s regression test to assess funnel plot asymmetry, which examines whether effect sizes tend to be larger in smaller studies. A *p*-value of less than 0.10 in Egger’s test was considered suggestive of significant publication bias.

## 3 Results

### 3.1 Study search

**Figure 1:**
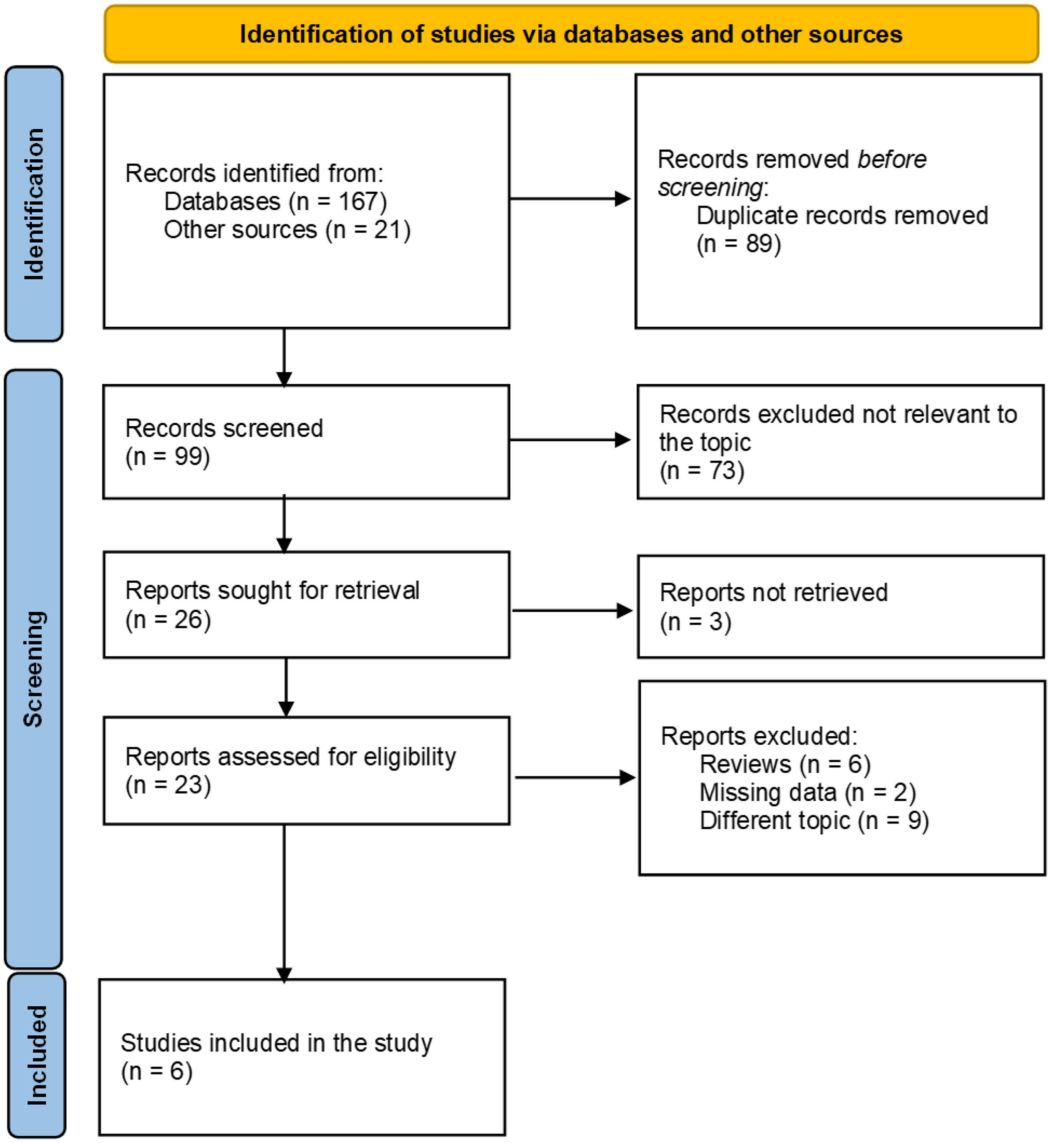
The flow diagram of included and excluded studies.

A total of 188 articles were initially identified through searches in PubMed, Embase, OVID, Web of Science, the CNKI, the Wanfang Database, Google Scholar, and Baidu Scholar. As illustrated in the flow diagram (Figure 1), 89 studies were excluded as duplicates retrieved from multiple databases. Following a title and abstract screening, 73 articles were removed due to lack of relevance to the CALLY index in the context of sepsis. The remaining 26 articles were sought for full-text retrieval, of which 3 were unavailable. Among the remaining 23 articles, 17 were excluded for being review articles or for not evaluating the value of the CALLY index. Consequently, six studies (10, 11, 15–18) comprising seven cohorts met the eligibility criteria and were included in the final meta-analysis.

### 3.2 Study characteristics

A total of 3,848 sepsis patients, comprising 2,870 survivors and 978 non-survivors, were included in this meta-analysis. As shown in Table 1, among the included studies, only two recruited patients from Turkey, while the participants in the remaining studies were all from China. Six cohorts from five studies analyzed the differences in CALLY index levels between sepsis patients and non-sepsis patients. Two studies reported the HR for the association between the CALLY index and mortality, and two studies reported the corresponding OR (Table 2).

**Table 1:** Characteristics of the study on the correlation between CALLY index and sepsis.

First author	Year	Country	During	Szie	M/F	Age	Type	Non-survivors			Survivors		SD
								N	Mean	SD	N	Mean	
Zhang et al. (11)	2024	China	2015.01–2023.11	1,123	707/416	74.67 ± 14.10 years	Sepsis	379	14.93	16.64	744	33.44	48.44
Yılmaz and Ak (10)	2025	Türkiye	2022.01–2024.01	669	290/379	64.40 ± 12.01 years	Sepsis	156	69.7	54.2	513	23.1	15.6
Lin et al. (15)	2025	China	2019.01–2024.06	105	79/26	68.78 ± 13.98 years	Septic shock	29	7.0	9.29	76	28.04	12.89
Lin et al. (15)	2025	China	2019.01–2024.06	38	24/14	63.90 ± 10.23 years	Septic shock	8	2.64	1.69	30	13.4	9.62
Sarıdaş et al. (16)	2025	Türkiye	2022.01–2025.01	1,644	906/738	69.27 ± 10.82 years	Sepsis	345	95.23	124.1	1,299	24.93	13.88
Wang et al. (17)	2025	China	2020.01–2024.12	143	77/66	49.57 ± 30.34 months	Sepsis	22	1.27	1.11	121	2.57	1.58

N, sample size; SD, standard deviation.

**Table 2:** The CALLY index in sepsis survivors versus non-survivors.

First author	Year	Univariate analysis			Multivariate analysis			Type	Diagnostic analysis			
		ES	LCI	UCI	ES	LCI	UCI		TP	FP	FN	TN
Zhang et al. (11)	2024	0.441	0.311	0.624	0.483	0.332	0.703	HR	181	217	198	527
Lai et al. (18)	2025	0	0	0.179	0.083	0.001	6.387	HR	NA	NA	NA	NA
Lin et al. (15)	2025	NA	NA	NA	0.96	0.92	0.99	OR	NA	NA	NA	NA
Wang et al. (17)	2025	0.47	0.287	0.771	0.532	0.323	0.877	OR	NA	NA	NA	NA
Yılmaz and Ak (10)	2025	NA	NA	NA	NA	NA	NA	NA	133	75	23	438

ES, effect size; LCI, lower limit of the confidence interval; UCI, upper limit of the confidence interval; HR, hazard ratio; OR, odds ratio; NA, not available; TP, true positive; FP, false positive; FN, false negative; TN, true negative.

### 3.3 Risk of bias results

Two reviewers independently evaluated the quality of the included studies using the NOS, with detailed results provided in Table 3. According to the NOS scores, one study (16.7%) was rated as being of moderate quality, as it did not achieve a full score in the comparability domain primarily due to inadequate control for key confounding factors and lack of adjustment for other relevant variables. The remaining studies (83.3%) were considered high quality.

**Table 3:** Quality assessment of includes studies using the Newcastlee-Ottawa scale.

Author	Year	Selection				Comparability		Exposure			Total
		1	2	3	4	1	2	1	2	3	
Zhang et al. (11)	2024	*	*	—	*	*	*	*	*	*	8
Yılmaz and Ak (10)	2025	*	*	—	*	*	—	*	*	*	7
Lin et al. (15)	2025	*	*	—	*	*	*	*	*	*	8
Lin et al. (15)	2025	*	*	—	*	*	*	*	*	*	8
Sarıdaş and Çetinkaya (16)	2025	*	*	—	*	—	*	*	*	*	7
Wang et al. (17)	2025	*	*	—	*	*	*	*	*	*	8
Lai et al. (18)	2025	*	*	—	*	—	—	*	*	*	6

Selection: 1. Case definition is adequate; 2. Consecutive or obviously representative series of cases; 3. Community controls; 4. No history of disease in controls; Comparability: 1. Study controls for the most important factor; 2. Comparability for any additional factor; Exposure: 1. Secure record/structured interview blind to case–control; 2. Same method for ascertainment for cases and controls status; 3. Non-response rate same for both groups; *: Asterisk means that the item is satisfied.

### 3.4 Differences in the CALLY index between survivors and non-survivors of sepsis

**Figure 2:**
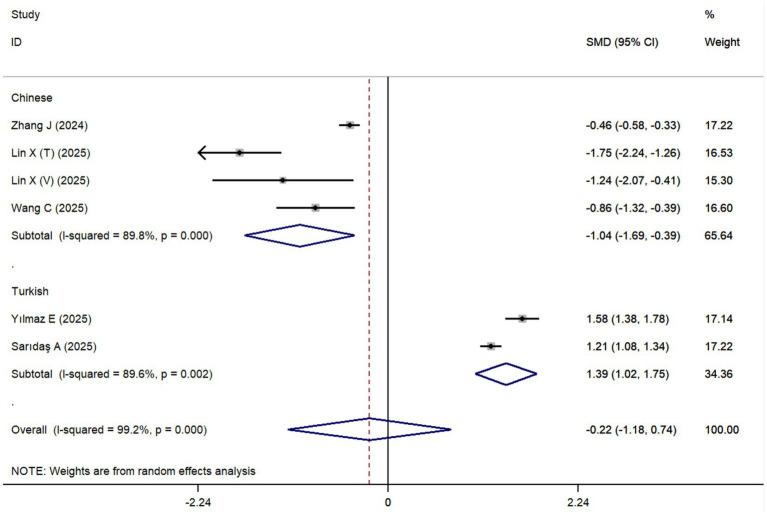
Differences in the CALLY index between survivors and non-survivors of sepsis.

A total of six cohort studies, comprising 3,722 sepsis patients (including 2,783 survivors and 939 non-survivors), were included to evaluate differences in the CALLY index between the two groups. The overall meta-analysis results indicated that there was no statistically significant difference in the CALLY index between survivors and non-survivors (SMD = −0.22, 95% CI: −1.18 to 0.74, *p* = 0.653). However, further subgroup analyses revealed that in the Chinese population, survivors had a significantly higher CALLY index than non-survivors (SMD = −1.04, 95% CI: −1.69 to −0.39, *p* = 0.002), while in the Turkish population, survivors had a significantly lower CALLY index (SMD = 1.39, 95% CI: 1.02 to 1.75, *p* < 0.001) (Figure 2). Substantial between-study heterogeneity was observed in both the Chinese and Turkish population subgroups (*I^2^* = 89.8 and 89.6%, respectively).

### 3.5 The CALLY index and mortality risk in sepsis

This meta-analysis examined the association between the CALLY index and mortality risk in patients with sepsis. The pooled results indicated that, in the univariate analysis, the combined HR was not statistically significant (HR = 0.05, 95% CI: 0.00–4.65, *p* = 0.295), whereas the combined OR showed a significant association (OR = 0.47, 95% CI: 0.29–0.73, *p* = 0.003). In contrast, in the multivariate analysis, the pooled HR demonstrated a statistically significant association (HR = 0.48, 95% CI: 0.33–0.69, *p* < 0.001), while the pooled OR was not statistically significant (OR = 0.75, 95% CI: 0.43–1.33, *p* = 0.332). Furthermore, substantial heterogeneity was observed in both the HR and OR pooled estimates across both univariate and multivariate analyses.

### 3.6 Diagnostic utility of the CALLY index in sepsis

A total of two studies evaluated the value of the CALLY index in predicting mortality in sepsis. The meta-analysis results demonstrated that the CALLY index had a sensitivity of 0.59 (95% CI: 0.54–0.63), a specificity of 0.77 (95% CI: 0.74–0.79), and a diagnostic odds ratio of 8.58 (95% CI: 0.59–124.59) for predicting sepsis mortality.

### 3.7 Publication bias and sensitivity analysis

**Figure 3:**
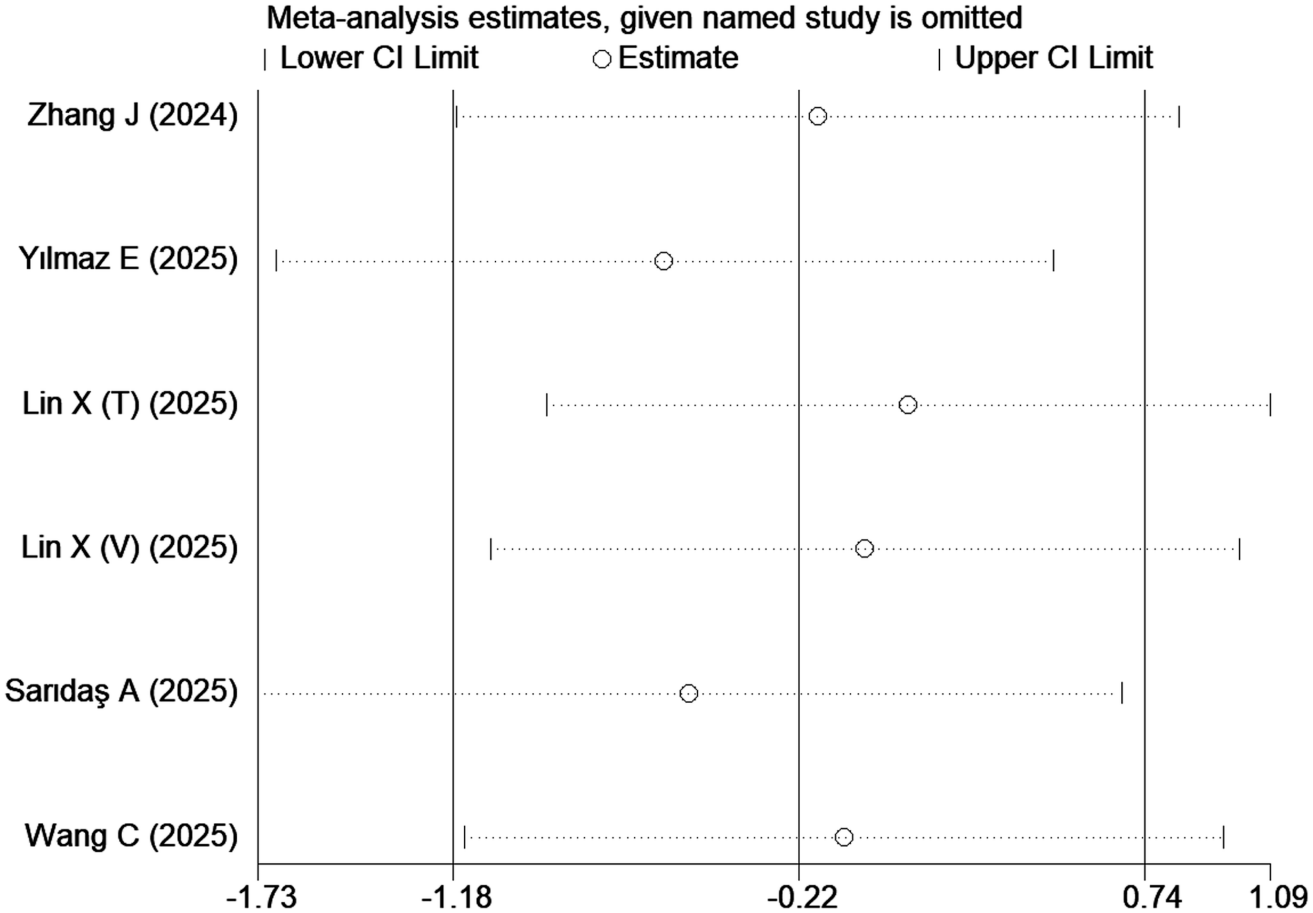
Sensitivity analysis of the association between the CALLY index and sepsis.

Publication bias was assessed for studies investigating differences in the CALLY index between sepsis survivors and non-survivors. Egger’s linear regression test showed a *p*-value of 0.550, suggesting no significant publication bias (Supplementary Figure S1). However, it should be noted that the reliability of statistical tests for publication bias is limited when the number of included studies is small, so the above result should be interpreted with caution. Furthermore, sensitivity analysis via the leave-one-out method confirmed the robustness of these pooled results (Figure 3).

## 4 Discussion

This study encompassing data from 3,848 sepsis patients, provides the first comprehensive synthesis of evidence regarding the prognostic and diagnostic value of the CALLY index in sepsis. The overall meta-analysis did not reveal a statistically significant difference in the CALLY index between survivors and non-survivors. However, subsequent subgroup analyses uncovered a striking divergence: a higher CALLY index was associated with improved survival in Chinese cohorts, whereas a lower index was linked to survival in the Turkish cohort. Furthermore, an elevated CALLY index suggests a correlation with reduced mortality risk in Chinese sepsis patients. Additionally, the index demonstrated moderate diagnostic accuracy for predicting mortality.

The most striking finding of this study is the opposing association of the CALLY index with mortality observed across different ethnic populations. This heterogeneity may stem from several factors. Firstly, ethnic variations in genetic background and host immune responses may influence the relationship between its components (CRP, albumin, lymphocytes) and the pathophysiology of sepsis. Secondly, differences in regional pathogen prevalence and antimicrobial resistance patterns could lead to variations in infection severity and the subsequent inflammatory and immune responses, which the CALLY index aims to capture. Thirdly, disparities in healthcare systems, including timing of patient presentation and intensive care unit admission standards, may also contribute to the observed divergent trend. Lastly, substantial measurement variability might exist in the retrospective inclusion of the CALLY index components; for instance, some studies might have included data from before the sepsis diagnosis, while others hours after diagnosis. This finding underscores the critical importance of considering geographic and ethnic factors when applying biomarker-based prognostic models in sepsis. Of course, our findings should be interpreted with caution. Given the limited number of included studies and potential imbalance in sample sizes across populations, the results of this meta-analysis should be considered exploratory rather than conclusive, and further validation is required in future research.

The CALLY index is a clinically attractive immuno-nutritional biomarker because it comprehensively reflects a patient’s inflammatory, nutritional, and immune status (19). Its major advantage stems from being calculated from inexpensive, readily available routine blood tests, making it particularly valuable for prognosis in resource-limited settings. Evidence supports the prognostic value of the CALLY index across various conditions. It has demonstrated superior predictive performance over conventional markers in cancers, including colorectal and non-small cell lung cancer (9, 20). Furthermore, studies have identified a linear negative correlation between the CALLY index and conditions like chronic obstructive pulmonary disease (21), and a lower CALLY score is associated with in-hospital mortality in heart failure patients, consistently indicating that a higher index predicts a better prognosis (22). Our findings are consistent with these studies, reinforcing that a higher CALLY index is associated with a better prognosis, thereby expanding its application scope. Furthermore, combining the CALLY index with established scores like SOFA shows promise in providing a more comprehensive assessment and improving prognostic accuracy for patients with septic shock (15).

In the present study, the observed high statistical heterogeneity, which persisted even within subgroup analyses, suggests that unmeasured confounding factors are likely influencing the value of the CALLY index. This phenomenon may be related to the following factors: Firstly, although all included patients had a definitive diagnosis of sepsis or septic shock, inconsistencies in their severity, comorbidities, and baseline characteristics existed. Secondly, although the SMD was used for pooled statistical analysis, the CALLY index itself varied across the included studies, which could still lead to bias in the results. Thirdly, the timing of the source for the components used to calculate the CALLY index may have been inconsistent among the included studies.

This meta-analysis represents the most comprehensive study in this field to date, thereby enhancing the reliability of its conclusions. However, certain limitations must be acknowledged when interpreting the results. Firstly, although subgroup and sensitivity analyses indicated that the overall findings were robust, significant heterogeneity was observed in some of the effect estimates, warranting caution in their interpretation. Secondly, many of the included studies were retrospective in design, which may introduce bias; thus, future large-scale prospective studies are required for validation. Thirdly, as the studies on the CALLY index in sepsis have all been published within the past year and the currently available published data are limited to China and Turkey, the conclusions may not be directly generalizable to other ethnic populations. Finally, our failure to search for unpublished studies or contact the original authors for missing data may increase the risk of publication bias.

## 5 Conclusion

In conclusion, this meta-analysis suggests that the CALLY index, as a novel, composite and low-cost prognostic biomarker, may hold potential for clinical application in patients with sepsis, particularly among Chinese populations. Future large-scale, multinational, prospective studies are warranted to validate its broad applicability and prognostic accuracy.

## Data Availability

The original contributions presented in the study are included in the article/Supplementary material, further inquiries can be directed to the corresponding authors.
