# Supplementary figures and images for: The prognostic value of the CALLY index in sepsis: a systematic review and meta-analysis
Source: Front Med (Lausanne). 2026 May 8;13:1812568. doi: 10.3389/fmed.2026.1812568 (PMC13237710; doi:10.3389/fmed.2026.1812568)

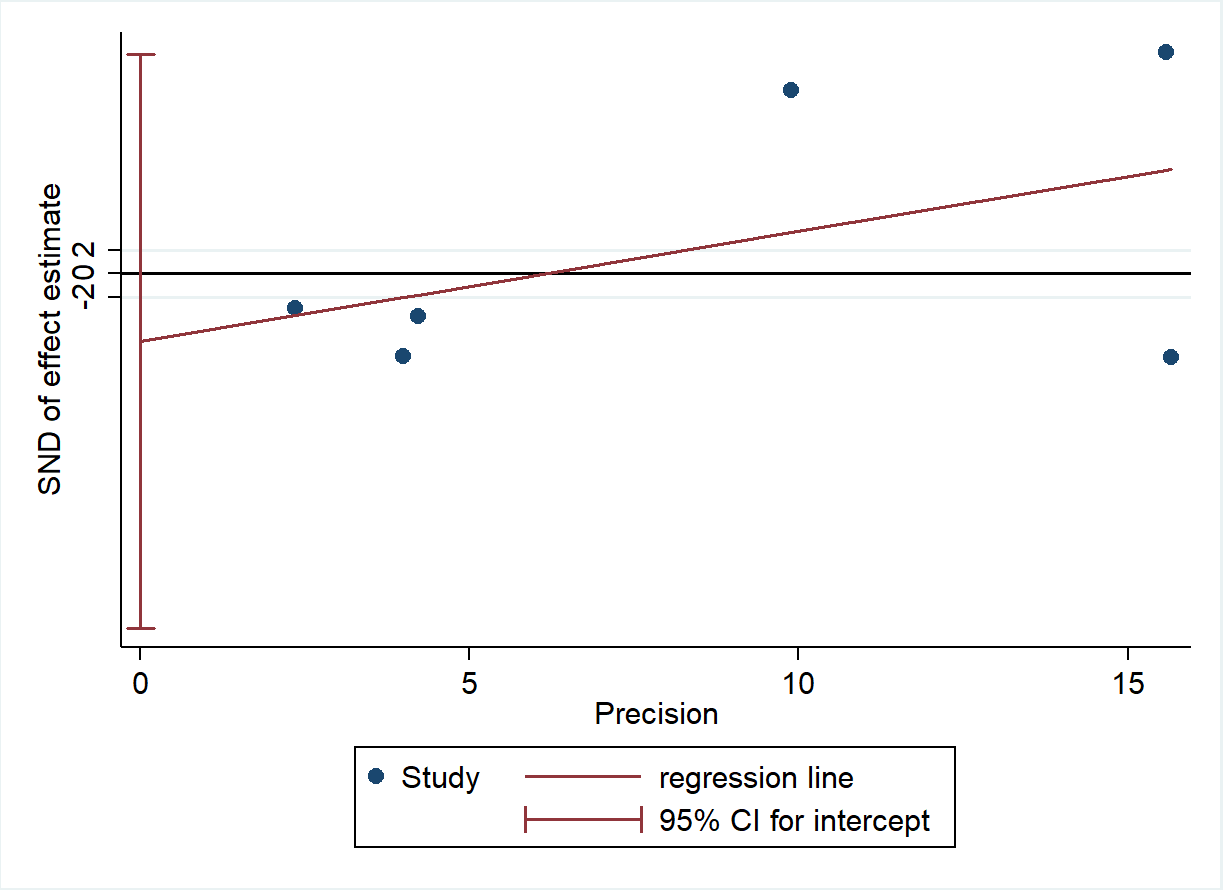

Supplement: Supplementary file 1 [file Image_1.TIF]
