# Supplementary material for: The prognostic value of the CALLY index in sepsis: a systematic review and meta-analysis
Source: Front Med (Lausanne). 2026 May 8;13:1812568. doi: 10.3389/fmed.2026.1812568 (PMC13237710; doi:10.3389/fmed.2026.1812568)
Supplement: Supplementary file 2 [file Supplementary_File_1.docx]

| **Datebase** | **Search strategy** |
| --- | --- |
| PUBMED | ((((Sepsis) OR (Bacteremia)) OR (Septic Shock)) OR (critically ill)) AND (((CRP-albumin-lymphocyte index) OR (CALLY index)) OR (C-reactive protein-albumin-lymphocyte index)) |
| Embase | (("Sepsis") OR ("Bacteremia") OR ("Septic Shock") OR ("critically ill")) AND (("CRP-albumin- lymphocyte index") OR ("CALLY index") OR ("C- reactive protein-albumin-lymphocyte index")) |
| OVID | ((Sepsis or Bacteremia or Septic Shock or  critically ill) and (CRP-albumin-  lymphocyte index or CALLY index or C-  reactive protein-albumin-lymphocyte  index)).af. |
| Web of Science | (Sepsis or Bacteremia or Septic Shock or critically ill) (Topic) and (CRP-albumin- lymphocyte index or CALLY index or C- reactive protein-albumin-lymphocyte index) (Topic) |
| CNKI and the Wanfang Database | CALLY AND (脓毒症 OR 脓毒性休克) |

Supplementary Table S1: Detailed search strategies
